# Clinical complexity in patients with atrial fibrillation: exploring differential risk profiles from European and Asian cohorts

**DOI:** 10.1093/europace/euaf229

**Published:** 2025-09-19

**Authors:** Andrea Galeazzo Rigutini, Tommaso Bucci, Michele Rossi, Enrico Tartaglia, Amir Askarinejad, Giulio Francesco Romiti, Cecilia Becattini, Giuseppe Boriani, Hung-Fat Tse, Tze-Fan Chao, Gregory Y H Lip

**Affiliations:** Liverpool Centre for Cardiovascular Science at University of Liverpool, Liverpool John Moores University and Liverpool Heart & Chest Hospital, Liverpool, United Kingdom; Internal, Vascular and Emergency Medicine—Stroke Unit, University of Perugia, Perugia, Italy; Liverpool Centre for Cardiovascular Science at University of Liverpool, Liverpool John Moores University and Liverpool Heart & Chest Hospital, Liverpool, United Kingdom; Liverpool Centre for Cardiovascular Science at University of Liverpool, Liverpool John Moores University and Liverpool Heart & Chest Hospital, Liverpool, United Kingdom; Department of Life, Health & Environmental Sciences, University of L'Aquila, L'Aquila, Italy; Internal Medicine and Nephrology Division, ASL1 Avezzano-Sulmona-L'Aquila, San Salvatore Hospital, L'Aquila, Italy; Liverpool Centre for Cardiovascular Science at University of Liverpool, Liverpool John Moores University and Liverpool Heart & Chest Hospital, Liverpool, United Kingdom; Cardiology Division, Department of Biomedical, Metabolic and Neural Sciences, Italy University of Modena and Reggio Emilia, Policlinico di Modena, Modena, Italy; Liverpool Centre for Cardiovascular Science at University of Liverpool, Liverpool John Moores University and Liverpool Heart & Chest Hospital, Liverpool, United Kingdom; Rajaie Cardiovascular Medical and Research Institute, Iran University of Medical Sciences, Tehran, Iran; Liverpool Centre for Cardiovascular Science at University of Liverpool, Liverpool John Moores University and Liverpool Heart & Chest Hospital, Liverpool, United Kingdom; Department of Traslational and Precision Medicine, Sapienza University of Rome, Rome, Italy; Internal, Vascular and Emergency Medicine—Stroke Unit, University of Perugia, Perugia, Italy; Cardiology Division, Department of Biomedical, Metabolic and Neural Sciences, Italy University of Modena and Reggio Emilia, Policlinico di Modena, Modena, Italy; The University of Hong Kong, Hong Kong, Hong Kong Special Administrative Region of China; Division of Cardiology, Department of Medicine, Taipei Veterans General Hospital, Taipei, Taiwan; Institute of Clinical Medicine and Cardiovascular Research Centre, National Yang Ming Chiao Tung University, Taipei, Taiwan; Liverpool Centre for Cardiovascular Science at University of Liverpool, Liverpool John Moores University and Liverpool Heart & Chest Hospital, Liverpool, United Kingdom; Department of Clinical Medicine, Aalborg University, Aalborg, Denmark; Department of Cardiology, Lipidology and Internal Medicine, Medical University of Bialystok, Bialystok, Poland

**Keywords:** Clinical complexity, Atrial fibrillation, Ethnic differences, Rhythm control, Oral anticoagulant

## Abstract

**Aims:**

Clinical complexity (CC) in atrial fibrillation (AF) reflects overlapping risk factors that raise vulnerability to both thromboembolism and bleeding. Ethnic differences in the expression of CC remain poorly characterized.

**Methods and results:**

We performed a post hoc analysis of the EORP-AF and APHRS-AF registries. CC was defined as a CHA₂DS₂–VASc score ≥2 plus ≥1 of: (i) age ≥75 and BMI <23 kg/m², (ii) chronic kidney disease, or (iii) prior major bleeding. Multivariable logistic regression identified predictors of CC, oral anticoagulant (OAC) use, and rhythm control. The primary outcome was a composite of all-cause death and major adverse cardiovascular events (MACE), defined as cardiovascular death, acute coronary syndromes, and thromboembolic events. Secondary outcomes included each individual component and major bleeding. Associations were assessed using Cox regression models. Among 14 055 patients, 2794 (19.9%) met CC criteria (mean age 77 ± 9 years; 46% female). Compared to Europeans, Asian patients with CC had a distinct clinical profile and were less likely to receive OAC (OR 0.75, 95% CI 0.57–1.01) or rhythm control (OR 0.53, 95% CI 0.41–0.69). CC was independently associated with increased risk of composite outcome (HR 1.55, 95% CI 1.35–1.77), all-cause death (HR 1.65, 95% CI 1.42–1.93), MACE (HR 1.50, 95% CI 1.26–1.80), cardiovascular death (HR 1.81, 95% CI 1.40–2.36), and major bleeding (HR 2.02, 95% CI 1.47–2.77). The excess risk of the composite outcome was greater in Asians (HR 2.28, 95% CI 1.57–3.32) than in Europeans (HR 1.51, 95% CI 1.31–1.75; *P*-interaction = 0.036).

**Conclusion:**

Among AF patients with CC, those enrolled in Asia exhibited marked differences in clinical profiles, management strategies, and outcomes, suggesting greater vulnerability to CC in the Asian population.

## Graphical Abstract

**Graphical Abstract:**
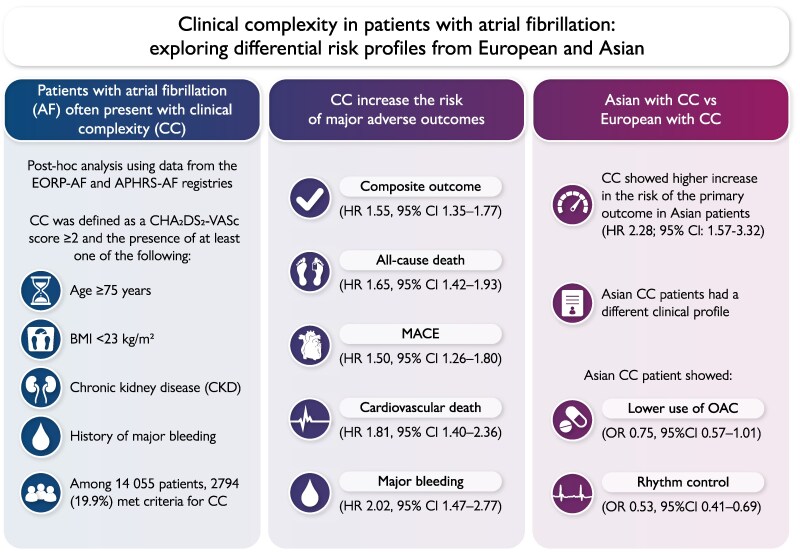

## Introduction

The global burden of atrial fibrillation (AF) is rising, as is the prevalence of older AF patients with multimorbidity, with consequent major implications for healthcare systems and patient outcomes.^1,2^ There are also geographical differences in the burden of AF, whereby Asians may have lower prevalence and incidence; nonetheless, given the size of Asian population, the numbers of AF patients far exceeds western populations^3^ and healthcare differences between different countries provide additional challenges in management and guideline implementation.^4,5^

Importantly, AF management is particularly challenging in patients who present with multiple, often competing, clinical risk factors. The concept of clinical complexity (CC) has been introduced^6,7^ to better identify this vulnerable subset of patients with AF, as they often require personalized and integrated management strategies. These strategies aim to account for the complexity of their condition and to determine appropriate therapeutic approaches that offer the best net clinical benefit, i.e. optimizing thrombotic prevention while minimizing bleeding risk.^8–10^

Despite increasing recognition of CC in patients with AF, limited data are available on how complexity differs across ethnic populations, and whether it influences clinical management and outcomes. For example, European and Asian patients with AF are known to differ in terms of body composition, bleeding and thrombotic risk, and therapeutic patterns, particularly regarding the use of oral anticoagulants (OACs).^11,12^ Yet, it remains unclear whether these differences translate into distinct complexity profiles or prognostic trajectories.

To address this knowledge gap, we analysed pooled data from two large international AF registries, the EURObservational Research Programme in Atrial Fibrillation General Long-Term Registry (EORP-AF; European cohort) and Asia–Pacific Heart Rhythm Society Atrial Fibrillation Registry (APHRS-AF; Asian cohort),^13,14^ with the following aims: (i) to determine the prevalence and clinical characteristics of CC across European and Asian cohorts; (ii) examine differences in management strategies among complex patients, including the use of anticoagulation and rhythm-control strategies; and (iii) evaluate the prognostic impact of CC on major adverse outcomes, with particular emphasis on differences related to patients’ enrolment settings.

## Methods

### Study design and population

This is a post hoc analysis of two large prospective observational registries: the EORP-AF and the APHRS-AF, which used a common protocol and study procedures, including standardized electronic case report form. Detailed protocols and baseline findings for both registries have been previously reported.^13,14^

Briefly, EORP-AF enrolled consecutive adult patients (≥18 years) with documented AF seen in inpatient or outpatient cardiology settings across 250 centres in 27 European countries between October 2013 and September 2016. The APHRS-AF registry recruited patients from 52 centres in Hong Kong, Japan, Singapore, South Korea, and Taiwan between 2015 and 2017. All patients underwent clinical evaluation by cardiologists and provided written informed consent prior to participation.

The EORP-AF registry was conducted in accordance with the European Union Note for Guidance on Good Clinical Practice (CPMP/ECH/135/95) and the Declaration of Helsinki. The APHRS-AF study protocol was approved by the local ethics committees of each participating site and was registered on ClinicalTrials.gov (NCT04807049).

### Data collection

Data were collected at baseline using a standardized electronic case report form common to both registries. Collected variables included demographics, medical history, comorbidities, AF characteristics, and pharmacological treatments. Follow-up was conducted prospectively for up to 2 years in EORP-AF and for 1 year in APHRS-AF, during which the occurrence of major adverse events—including thromboembolic events (TEE), cardiovascular events, bleeding, and mortality—was systematically recorded.

### Cohort and definitions

CC was defined as previously described,^7,9^ as a CHA₂DS₂–VASc score ≥2 in combination with at least one of the following criteria:

(i) Age ≥75 years and body mass index (BMI) < 23 kg/m², consistent with a BMI cut-off previously identified among elderly individuals^15^;
(ii) Chronic kidney disease (CKD), defined as a documented history of renal impairment or estimated glomerular filtration rate (eGFR) < 60 mL/min/1.73 m²;
(iii) History of major bleeding (MB), defined as any clinically relevant haemorrhagic event prior to enrolment, as documented by the investigator.

Rhythm control strategy was defined as the administration of Class Ia, Class Ic, or Class III antiarrhythmic drugs, or the performance of electrical or pharmacological cardioversion, or catheter ablation.

Thromboembolic risk was assessed using the CHA₂DS₂–VASc score,^16^ whereas the bleeding risk was assessed with the HAS-BLED score.^17^ OACs use was defined based on prescription at baseline of either vitamin K antagonists or direct OACs. According to guidelines contemporaneous to patient enrolment, OAC therapy was recommended for men with CHA₂DS₂–VASc ≥1 and women with CHA₂DS₂–VASc ≥2.^18,19^

Patients were stratified according to the presence or absence of CC, and for the purpose of this analysis, we included only patients with complete data on CC use and follow-up information.

### Outcomes

The primary outcome was a composite of all-cause death and major adverse cardiovascular events (MACE). MACE was defined as a combination of cardiovascular death (CV death), any acute coronary syndrome (ACS), and TEE.

All-cause mortality encompassed death from cardiovascular, non-cardiovascular, or undetermined causes. Cardiovascular death was defined as death resulting from ACS, heart failure (HF), arrhythmia, cardiac perforation, tamponade, or other unspecified cardiac conditions.

Secondary outcomes included the individual components of the primary endpoint (all-cause mortality and MACE) as well as MB events. MB was defined as the occurrence of intracranial haemorrhage (ICH) or major extracranial bleeding. Major extracranial bleeding was defined as any bleeding event leading to a haemoglobin decrease >2 g/dL, requiring blood transfusion, or resulting in hospitalization, involving any major organ system.

### Statistical analysis

Continuous variables were reported as mean ± standard deviation and compared using Student’s *t* test. Categorical variables were summarized as counts (percentages) and compared using the χ² test.

Descriptive comparisons were performed at two levels: (i) CC vs. non-CC patients, and (ii) CC patients stratified by geographic cohort (Asian vs. European).

Univariable and multivariable logistic regression analyses were used to identify factors associated with: (i) recruitment in Asia vs. Europe, within the CC population, (ii) OAC prescription, and (iii) use of rhythm-control strategies. Results were expressed as odds ratios (ORs) with 95% confidence intervals (CIs). Multivariable models were adjusted for the following covariates: age, female sex, BMI, hypertension, diabetes mellitus, dyslipidaemia, smoking status, dementia, HF, coronary artery disease (CAD), peripheral artery disease (PAD), prior MB, CKD, prior TEE, chronic obstructive pulmonary disease (COPD), cancer, and paroxysmal AF.

Incidence rates of clinical outcomes—including all-cause death, MACE, CV death, ACS, TEE, MB, and composite outcomes—were calculated and expressed as events per 100 person-years, with exact Poisson 95% CIs and between-group comparisons using the Poisson test.

Univariable and multivariable Cox proportional hazards regression analyses were used to estimate hazard ratios (HRs) and 95% CIs for the risk of adverse clinical outcomes in CC patients compared with non-CC patients. Separate multivariable Cox models were constructed for the primary and secondary outcomes, adjusting for age, BMI, female sex, paroxysmal AF, HF, hypertension, diabetes, TEE, PAD, CAD, cancer, dementia, use of OACs, and cohort of recruitment (Asia vs. Europe). The proportional hazards assumption was assessed using Schoenfeld residuals. Kaplan–Meier survival curves were constructed for all clinical outcomes and compared between CC and non-CC patients using the log-rank test.

Subgroup analyses were performed to evaluate potential effect modification of CC across clinically relevant strata, including: age (<75 or ≥75 years), sex (male or female), cohort of recruitment [Asian (APHRS) vs. European (EORP)], BMI (<23 or ≥23 kg/m²), smoking status, hypertension, PAD, CAD, diabetes, HF, dementia, prior thromboembolism, cancer, CKD, and OAC use. Results of the subgroup analyses were reported graphically, with HRs and interaction *P*-values.

All statistical analyses were performed using R version 4.3.1 (R Foundation for Statistical Computing, Vienna, Austria). A two-sided *P*-value <0.05 was considered statistically significant.

## Results

### Baseline characteristics

Among 14 055 patients with AF, 2794 (20%) met the criteria for CC. Compared with non-CC patients, those with CC were older (77 ± 9 vs. 67 ± 11; *P* < 0.001), more often female, and had significantly lower BMI. Patients with CC showed a broad spectrum of comorbidities—including cardiovascular, renal, metabolic, oncologic, and cognitive conditions—which were significantly more prevalent among CC patients (*Table 1*). History of bleeding was more prevalent among CC patients, with higher rates of prior ICH, major extracranial bleeding, and clinically relevant non-MB (*all P* < 0.001). A history of systemic TEE was more common in patients with CC, primarily driven by ischaemic stroke (*all P* < 0.001; *Table 1*). Lastly, CC patients were less likely to present with paroxysmal AF and showed a lower use of rhythm-control strategies compared to AF patients without CC (both *P* < 0.001). Despite their higher thromboembolic risk, prevalence of OAC use was slightly lower in patients with CC (83% vs. 85%, *P* = 0.002).

**Table 1:** Baseline characteristics of patients with clinical complexity and non-clinical complexity and baseline characteristics of Asian patients with clinical complexity and European with clinical complexity

Characteristic	Non-clinical complexity *N* = 11,261*^a^*	Clinical complexity *N* = 2,794*^a^*	*P*-value*^b^*	European with clinical complexity *N* = 1,936*^a^*	Asian with clinical complexity *N* = 858*^a^*	*P*-value*^b^*
Age, mean ± SD (years)	67 ± 11	77 ± 9	<0.001	76 ± 8	78 ± 9	<0.001
Sex			<0.001			0.058
Male	7076 (63%)	1519 (54%)		1029 (53%)	490 (57%)	
Female	4185 (37%)	1275 (46%)		907 (47%)	368 (43%)	
BMI	27.9 ± 5.0	25.6 ± 5.4	<0.001	26.8 ± 5.5	22.8 ± 4.1	<0.001
Systolic pressure	132 ± 20	133 ± 21	0.036	133 ± 21	131 ± 20	0.014
Diastolic pressure	79 ± 12	76 ± 13	<0.001	78 ± 13	72 ± 12	<0.001
Heart rate	81 ± 22	81 ± 22	0.3	83 ± 23	76 ± 17	<0.001
Hypertension	6584 (59%)	2021 (73%)	<0.001	1384 (72%)	637 (75%)	0.2
Diabetes mellitus	2319 (21%)	911 (33%)	<0.001	637 (33%)	274 (32%)	0.7
Lipid disorder	4250 (39%)	1241 (46%)	<0.001	863 (46%)	378 (45%)	0.5
Smoking	1046 (10%)	144 (5.6%)	<0.001	99 (5.5%)	45 (5.7%)	>0.9
Heart failure	3293 (30%)	1433 (52%)	<0.001	1147 (60%)	286 (34%)	<0.001
Valvular disease	5069 (46%)	1770 (65%)	<0.001	1230 (65%)	540 (65%)	>0.9
Coronary artery disease	2436 (23%)	959 (36%)	<0.001	734 (41%)	225 (27%)	<0.001
Peripheral vascular disease	539 (4.9%)	289 (11%)	<0.001	269 (14%)	20 (2.3%)	<0.001
Pulmonary arterial hypertension	597 (5.4%)	305 (11%)	<0.001	233 (12%)	72 (8.4%)	<0.001
Aortic plaque	283 (2.5%)	165 (6.0%)	<0.001	145 (7.5%)	20 (2.4%)	<0.001
COPD	703 (6.3%)	298 (11%)	<0.001	254 (13%)	44 (5.2%)	<0.001
CKD	81 (0.7%)	1453 (52%)	<0.001	1180 (61%)	273 (32%)	<0.001
Prior ischaemic stroke	628 (5.6%)	261 (9.4%)	<0.001	169 (8.7%)	92 (11%)	<0.001
Prior thromboembolic events	1131 (10%)	468 (17%)	<0.001	316 (17%)	152 (18%)	0.4
Cancer	176 (1.6%)	113 (4.1%)	<0.001	73 (3.8%)	40 (4.7%)	0.3
Dementia	98 (0.9%)	108 (3.9%)	<0.001	71 (3.7%)	37 (4.3%)	0.5
Paroxysmal Atrial fibrillation	3520 (32%)	731 (26%)	<0.001	393 (21%)	338 (39%)	<0.001
Intracranial haemorrhage	24 (0.8%)	146 (11%)	<0.001	88 (19%)	58 (6.8%)	<0.001
Prior major extracranial bleeding	42 (1.3%)	335 (25%)	<0.001	228 (48%)	107 (13%)	<0.001
Clinically relevant non-major bleeding	33 (1.0%)	328 (25%)	<0.001	209 (44%)	119 (14%)	<0.001
Aspirin or other antiplatelet drugs	2288 (20%)	803 (29%)	<0.001	542 (28%)	261 (30%)	0.4
CHA₂DS₂−VASc score	2.70 ± 1.70	4.22 ± 1.42	<0.001	4.31 ± 1.46	4.01 ± 1.31	<0.001
HAS-BLED ≥ 3	998 (8.9%)	1288 (46%)	<0.001	974 (50%)	314 (37%)	<0.001
VKAs	4600 (41%)	1225 (44%)	0.005	1023 (53%)	202 (24%)	<0.001
NOACs	5029 (45%)	1100 (39%)	<0.001	600 (31%)	500 (58%)	<0.001
OACs	9621 (85%)	2324 (83%)	0.002	1622 (84%)	702 (82%)	0.2
Rhythm control	3806 (40%)	692 (28%)	<0.001	495 (30%)	197 (25%)	0.017
Group			0.002			
European	8141 (72%)	1936 (69%)				
Asian	3120 (28%)	858 (31%)				

AF, atrial fibrillation; BMI, body mass index; CKD, chronic kidney disease; COPD, chronic obstructive pulmonary disease; NOAC, non-vitamin K antagonist anticoagulant; OACs, oral anti-coagulants; VKA, vitamin K antagonist.

^a^Mean ± SD; *n* (%).

^b^Welch Two sample *t*-test; Pearson's Chi-squared test.

### Survival analysis

Among the 2794 patients with CC, and after a median follow-up of 694 days (IQR, 365–734), the composite outcome occurred in 572 (20.5%), while all-cause death in 456 (16.3%), MACE in 303 patients (10.8%), CV death in 167 (6.0%), ACS in 82 (2.9%), VTE in 71 (2.5%), and MB in 92 (3.3%) (*Table 2*).

**Table 2:** Incidence rates and Cox regression analyses for risk of primary and secondary outcomes according to clinical complexity

	Number of events	Incidence rate per 100 patient-years (95% CI)	*P*-value	Univariable analysis HR (95% CI)	Multivariable analysis HR (95% CI)
Composite outcome					
Clinical complexity	572	14.6 (13.4–15.8)	<0.0001	2.76 (2.48–3.06)	1.55 (1.35–1.77)
Non-clinical complexity	927	5.2 (4.9–5.6)		Reference	Reference
All-cause death					
Clinical complexity	456	11.2 (10.2–12.3)	<0.0001	3.32 (2.94–3.75)	1.65 (1.42–1.93)
Non-clinical complexity	613	3.4 (3.1–3.7)		Reference	Reference
MACE					
Clinical complexity	303	7.7 (6.9–8.7)	<0.0001	2.47 (2.14–2.85)	1.50 (1.26–1.80)
Non-clinical complexity	553	3.1 (2.8–3.4)		Reference	Reference
CV Death					
Clinical complexity	167	4.1 (3.5–4.8)	<0.0001	3.44 (2.81–4.21)	1.81 (1.40–2.36)
Non-clinical complexity	214	1.8 (1.0–1.3)		Reference	Reference
ACS					
Clinical complexity	82	2.1 (1.5–2.5)	<0.0001	1.85 (1.43–2.40)	1.32 (0.96–1.82)
Non-clinical complexity	196	1.1 (0.9–1.3)		Reference	Reference
Thromboembolic events					
Clinical complexity	71	1.8 (1.4–2.3)	<0.0001	1.81 (1.37–2.38)	1.36 (0.96–1.92)
Non-clinical complexity	176	1.0 (0.9–1.2)		Reference	Reference
Major bleeding					
Clinical complexity	92	2.3 (1.9–2.9)	<0.0001	2.65 (2.04–3.43)	2.02 (1.47–2.77)
Non-clinical complexity	154	0.9 (0.7–1.0)		Reference	Reference

ACS, acute coronary syndrome; CV death, cardiovascular death; MACE, major adverse cardiovascular events.

**Figure 1:**
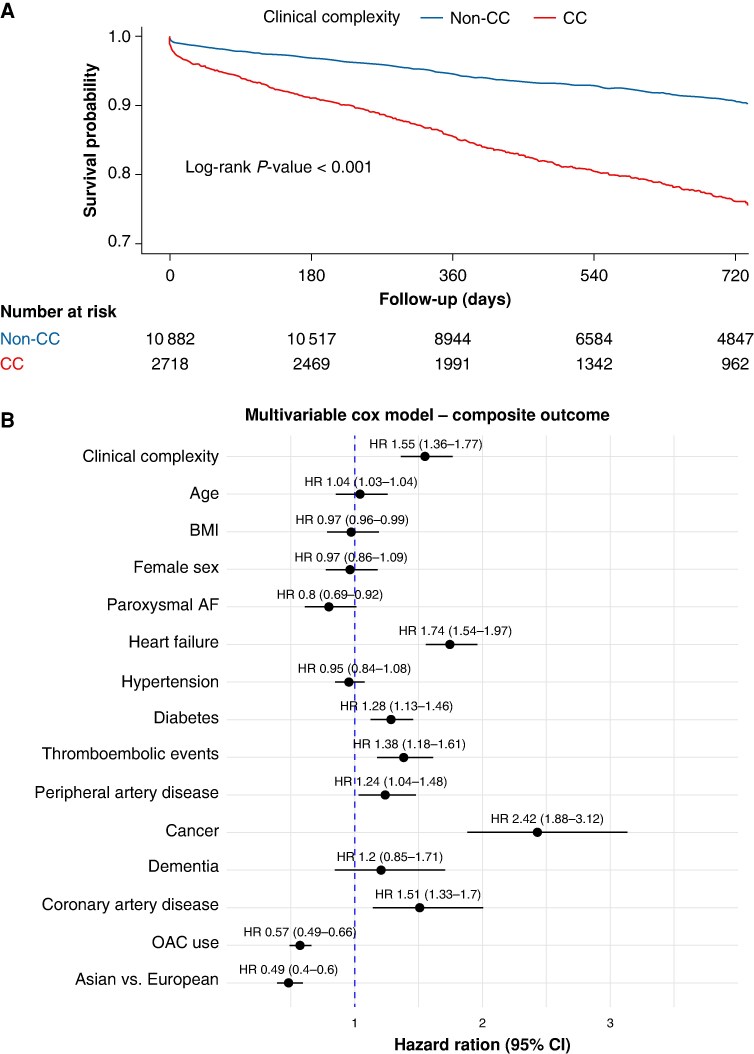
Kaplan–Meier curves and multivariable Cox analysis for the composite outcome according to clinical complexity. Panel A shows Kaplan–Meier curves for the composite outcome of all-cause death and major adverse cardiovascular events (MACE) stratified by presence of clinical complexity. Panel B shows hazard ratios (HRs) from the multivariable Cox regression model including demographic and clinical covariates. AF, atrial fibrillation; BMI, body mass index; CC, clinical complexity group; OAC, oral anticoagulant.

**Figure 2:**
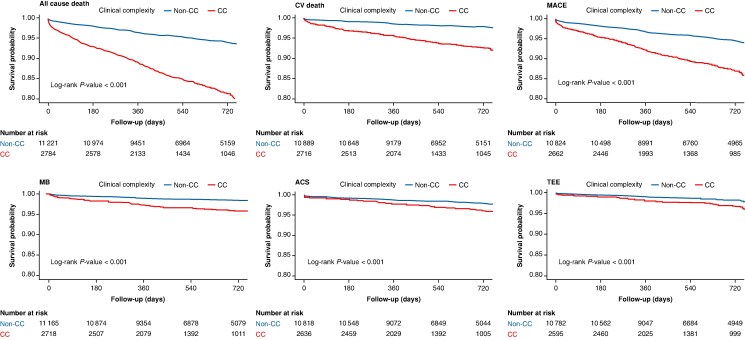
Kaplan–Meier curves for the secondary outcomes according to clinical complexity. Kaplan–Meier curves for secondary outcomes including all-cause death, cardiovascular death, major adverse cardiovascular events, MB, ACS, and thromboembolic events, stratified by presence of clinical complexity. ACS, acute coronary syndrome; CC, clinical complexity group; CV death, cardiovascular death; CV, cardiovascular; MACE, major adverse cardiovascular events; MB, major bleeding; VTE, venous thromboembolism.

CC patients showed higher incidence rates of both primary and secondary outcomes, compared to non-CC patients (*Table 2*). On univariable Cox regression analysis (*Table 2*, *Figures 1A* and *2*), compared with non-CC patients, those with CC had a higher risk of composite outcome, all-cause death, MACE, CV death, ACS, TEE, and MB. At multivariable analysis, CC remained independently associated with a higher risk of the composite outcome (HR 1.55; 95% CI 1.35–1.77), all-cause death (HR 1.65; 95% CI 1.42–1.93), MACE (HR 1.50; 95% CI 1.26–1.80), CV death (HR 1.81; 95% CI 1.40–2.36), and MB (HR 2.02; 95% CI 1.47–2.77) (*Table 2*). In contrast, the association with ACS (HR 1.32; 95% CI 0.96–1.82) and TEE (HR 1.36; 95% CI 0.96–1.92) were of lower magnitude compared to the unadjusted analysis, and not statistically significant due to wide 95% CIs (*Table 2*).

Regarding potential differences related to the enrolment setting, recruitment in Asia was associated with a significantly lower risk of composite outcome (HR 0.49; 95% CI 0.40–0.60) (*Figure 1B*), all-cause death (HR 0.50; 95% CI 0.39–0.64), CV death (HR 0.32; 95% CI 0.20–0.53), ACS (HR 0.38; 95% CI 0.23–0.63), and VTE (HR 0.58; 95% CI 0.36–0.92) compared to those enrolled in the European cohort (see Supplementary material online, *Table S1*).

### Differences between European and Asian patients with clinical complexity

Among the 2794 patients with CC, 1936 (69%) were recruited in the EORP-AF registry, and 858 (31%) in the APHRS-AF registry. Patients recruited in Asia were older (78 ± 9 vs. 76 ± 8; *P* < 0.001), with lower BMI (22.8 ± 4.1 vs. 26.8 ± 5.5 kg/m²; *P* < 0.001), lower diastolic blood pressure, and lower heart rate, when compared to patients recruited in Europe. Asian patients more commonly showed paroxysmal AF (39% vs. 21%; *P* < 0.001), but they were also less frequently managed with rhythm-control strategies (25% vs. 30%; *P* = 0.017). Conversely, patients recruited in Europe exhibited a higher cardiovascular burden, with higher prevalences of HF (60% vs. 34%; *P* < 0.001), CAD, PAD, pulmonary arterial hypertension, aortic plaque, COPD (13% vs. 5.2%; *P* < 0.001), and CKD. European patients had a higher risk of bleeding, as reflected by a greater proportion of patients with HAS-BLED scores ≥3 (50% vs. 37%; *P* < 0.001). Additional clinical and therapeutic differences between the two groups are summarized in *Table 1*.

Results of the univariable and multivariable logistic regression models are reported in Supplementary material online, *Table S2*. Patients in the Asian cohort were younger (OR per year increase 0.98, 95% CI 0.97–1.00), less commonly female (OR 0.56, 95% CI 0.44–0.70), and had lower BMI (OR per unit increase 0.84, 95% CI 0.81–0.86); however, they were more likely to have paroxysmal AF (OR 2.12, 95% CI 1.68–2.67), diabetes mellitus (OR 1.76, 95% CI 1.38–2.25), dyslipidaemia (OR 1.36, 95% CI 1.08–1.71), and hypertension (OR 1.46, 95% CI 1.15–1.87). Patients recruited in Asia showed also lower odds of presenting with CKD (OR 0.56, 95% CI 0.44–0.71), HF (OR 0.54, 95% CI 0.43–0.68), CAD (OR 0.67, 95% CI 0.52–0.85), PAD (OR 0.17, 95% CI 0.10–0.30), and COPD (OR 0.46, 95% CI 0.30–0.69) at baseline.

### Factors associated with oral anticoagulant use in clinical complexity patients

Results of the univariable and multivariable regression models on the association of baseline characteristics with use of OAC in patients with CC are reported in Supplementary material online, *Table S3*. At multivariable analysis, presence of CAD (OR 0.60, 95% CI 0.46–0.78), HF (OR 0.77, 95% CI 0.59–0.99), dementia (OR 0.43, 95% CI 0.25–0.77), cancer (OR 0.25, 95% CI 0.16–0.40), and paroxysmal AF (OR 0.66, 95% CI 0.51–0.87) were associated with lower odds of OAC use at baseline, with a non-significant trend observed also for female sex (OR 0.81, 95% CI 0.63–1.01), and recruitment in Asia (OR 0.75, 95% CI 0.57–1.01). Conversely, a history of TEE was associated with increased odds of receiving OACs (OR 1.65, 95% CI 1.16–2.41).

### Factors associated with rhythm control strategy in clinical complexity patients

Full results of the univariable and multivariable regression on use of rhythm control strategy in patients with CC are shown in Supplementary material online, *Table S4*. On multivariable logistic regression, older age (OR per year increase 0.97, 95% CI 0.96–0.99), diabetes mellitus (OR 0.62, 95% CI 0.49–0.80), smoking (OR 0.52, 95% CI 0.29–0.90), HF (OR 0.77, 95% CI 0.59–0.99), CAD (OR 0.64, 95% CI 0.51–0.80), dementia (OR 0.57, 95% CI 0.25–0.90), and recruitment in Asia (OR 0.53, 95% CI 0.41–0.69) were all associated with lower odds of receiving rhythm control. Conversely, paroxysmal AF was associated with higher likelihood of receiving rhythm control approaches (OR 2.53, 95% CI 2.02–3.18).

### Subgroup analysis

**Figure 3:**
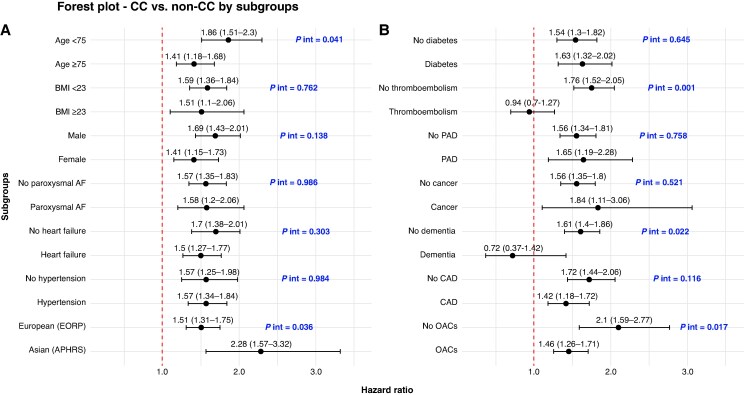
Effect of clinical complexity on the composite outcome in prespecified subgroups. Forest plot showing hazard ratios for the association between clinical complexity and the composite outcome in prespecified subgroups defined by demographic and clinical characteristics. AF, atrial fibrillation; BMI, body mass index; CKD, chronic kidney disease; COPD, chronic obstructive pulmonary disease; NOAC, non-vitamin K antagonist anticoagulant; OACs, oral anti-coagulants; PAD, peripheral artery disease; VKA, vitamin K antagonist; CAD, coronary artery disease.

The impact of CC on the risk of the composite outcome was greater in Asian patients compared to European patients (HR 2.28; 95% CI 1.57–3.32 vs. HR 1.51; 95% CI 1.31–1.75, respectively; *P* for interaction = 0.036), as well as in patients aged <75 years (*P* for interaction = 0.041), those without prior TEE (*P* for interaction = 0.001), those not treated with OACs (*P* for interaction = 0.017), and in patients without dementia (*P* for interaction = 0.022) (*Figure 3*).

## Discussion

In this study, our principal findings are as follows: (i) CC was present in approximately 20% of the population and was associated with a higher burden of cardiovascular, renal, oncologic, and cognitive comorbidities; (ii) Asian patients with CC had distinct clinical profiles and were less commonly prescribed OAC and rhythm control strategies compared to Europeans CC patients; and (iii) CC was associated with an increased risk of adverse events. While European CC patients had a higher absolute risk of adverse outcomes compared to Asian CC patients, the relative impact of CC appeared to be significantly greater among Asian patients.

The 20% prevalence of CC observed in our study is slightly lower than that reported in the GLORIA-AF registry, where a CC phenotype was identified in approximately 32% of patients.^7^ Nonetheless, these data highlight that CC affects a substantial proportion of the AF population and represents an increasingly relevant clinical issue. Indeed, beyond the general increase in AF incidence, the evolving burden of comorbidities, ageing, and systemic frailty has reshaped the clinical profile of AF patients, leading to the emergence of a particularly vulnerable subgroup often defined as ‘clinically complex’.^20^

The CC subgroup cannot be overlooked, as they embody a convergence of competing risks that complicate management and are associated with poorer outcomes. In line with this, our data showed that CC patients were significantly older, more often female, and carried a greater burden of cardiovascular, renal, oncologic, and cognitive comorbidities. Additionally, a history of bleeding was over ten times more common among CC patients compared to those without CC, yet anticoagulation rates differed only marginally. Similarly, rhythm-control strategies were used less frequently, suggesting a degree of therapeutic hesitancy in the context of perceived CC.

Despite growing recognition of CC, its geographic and ethnic variability remains largely unexplored. In our study, Asian patients with CC exhibited a markedly different clinical and physiological profile compared to their European counterparts. They had significantly lower BMI, more frequent paroxysmal AF, lower resting heart rate, and overall lower CHA₂DS₂–VASc scores. In contrast, European patients showed a higher prevalence of cardiovascular and systemic comorbidities—including HF, vascular disease, CKD, prior bleeding, and COPD—suggesting a more advanced stage of vascular ageing and systemic frailty.

These divergent clinical profiles are broadly consistent with data from the GARFIELD-AF registry,^21^ which showed that Asian patients had lower BMI, a higher prevalence of paroxysmal AF, lower heart rate, and lower thromboembolic risk scores compared to non-Asian participants, whereas European patients more frequently exhibited vascular disease, prior bleeding, and renal impairment. While in GARFIELD-AF, the prevalence of HF was only marginally higher among Europeans, our data show more pronounced differences, with nearly double the burden of HF in the European CC population. Additionally, we observed a higher prevalence of COPD among Europeans, which may further complicate the clinical management of these patients.^22,23^

These clinical differences were mirrored by distinct treatment patterns. Asian patients with CC were less likely to receive OACs compared to their European counterparts.^24^ While this discrepancy may reflect non-clinical determinants, such as healthcare system characteristics, reimbursement policies, or drug availability, as previously reported,^25–30^ it may also relate to clinical factors, including a higher perceived or actual bleeding risk that contraindicates OAC use. A similar trend was observed for rhythm control strategies, which were also less frequently adopted among Asian patients with CC.^29,31,32^ In this context, beyond healthcare system aspects, another possible explanation may lie in the higher prevalence of symptom-controlled AF among Asian patients, which could have led to a lower use of rhythm control interventions—particularly considering that this study was conducted before evidence became available showing a prognostic benefit of rhythm-control over rate-control strategies.^33,34^

Nevertheless, it is unlikely that these treatment differences can be accounted for solely by clinical characteristics; rather, they appear to be driven by system-level, socioeconomic, and contextual determinants. In particular, disparities in healthcare resources and the unequal availability or reimbursement of DOACs across regions have been associated with restricted access to optimal anticoagulation,^26,27^ underscoring the role of healthcare system organization and broader contextual factors in shaping geographic heterogeneity in management patterns.

This scenario becomes even more complex when considering the marked prognostic differences observed across geographical regions in our study, with European CC patients exhibiting a consistently higher risk of adverse outcomes compared to Asian CC patients. This underscores the notion that CC reflects intrinsic biological and systemic vulnerability, rather than merely serving as a marker of undertreatment, thereby confirming findings from previous large cohort studies.^35,36^ While plausible explanations for the prognostic differences may include the greater burden of cardiovascular risk factors among European patients^37^ and the higher prevalence of paroxysmal AF among Asian patients. These findings underscore the need for particular attention to ethnic and racial differences when stratifying prognostic risk and establishing appropriate therapeutic strategies in CC patients. Indeed, population differences in the clinical epidemiology of AF,^38^ as well as AF-related complications (including stroke and bleeding)^39,40^ have been reported. In this context, recent evidence further demonstrates that racial differences extend to bleeding risk patterns in AF, thereby reinforcing the need for race-specific considerations when assessing prognosis and tailoring management strategies.^41^

Despite differences in the overall risk of adverse events across CC populations, our subgroup analysis revealed that the prognostic impact of CC was significantly greater in Asian patients than in Europeans. This suggests that when CC develops in Asian patients, it may reflect a more substantial shift in clinical vulnerability. A plausible explanation for this differential effect is the generally lower baseline risk of thrombotic and haemorrhagic events among Asian patients compared with their European counterparts. Against this lower-risk background, the onset of CC marks a transition from a relatively low-risk profile to a distinctly vulnerable state, thereby producing a steeper relative risk gradient once CC is present. Conversely, in patients with higher baseline clinical risk, as seen in our European cohort, the relative impact of CC may be less pronounced. Moreover, the combination of younger age and lower use of OACs in Asian patients with CC, two factors that according to our subgroup analysis amplify the prognostic impact of CC, likely contributes to the more abrupt deterioration observed when complexity arises in this population.

These findings challenge the conventional notion that younger age is protective in AF. Recent data from a large contemporary cohort of over 17 000 patients with AF aged <65 years demonstrated a substantial burden of comorbidities in this population, translating into markedly increased risks of long-term mortality and cardiovascular hospitalizations.^42^ Therefore, in the context of CC, younger age may not provide the expected prognostic advantage. Rather, the accumulation of comorbidities in younger individuals may signal an early shift toward a vulnerable clinical phenotype, in which the relative impact of CC is accentuated. These observations highlight the limitations of relying solely on chronological age for risk stratification in AF, and reinforce the need for a more integrated, phenotype-based approach that accounts for the broader context of CC.

A greater susceptibility to CC was observed among patients not receiving OACs at baseline. This may be explained by the accumulation of additional bleeding risk factors—such as cancer, dementia, complex antithrombotic regimens, and a history of prior bleeding—which may have contraindicated OAC use and thereby further increased the already elevated intrinsic risk of adverse outcomes in CC patients. Indeed, previous studies have consistently reported high rates of OAC discontinuation among AF patients with CC, accompanied by a rise in both thrombotic and haemorrhagic events compared to those who remained on anticoagulation.^7,9^ These findings underscore the complexity of evaluating the net clinical benefit of OAC therapy in complex populations, despite the well-established safety and efficacy profile of non-vitamin K antagonist OACs.^7,43^

These insights reinforce the notion that CC does not exert a uniform effect across patient subgroups, but rather interacts with baseline risk phenotypes to shape distinct outcome trajectories. Our study expands upon existing literature by demonstrating both the prevalence and the prognostic impact of CC across European and Asian populations. Collectively, these findings underscore that the clinical manifestation of complexity in AF is not homogeneous, but instead influenced by regional, phenotypic, and health system factors. Recognizing these variations is essential to refine risk stratification models and to develop context-specific strategies for optimal patient care, in line with the holistic framework promoted by recent evidence-based integrated approaches to AF management,^44–46^ as recommended by the latest guidelines.^47,48^

### Limitations

This study has several limitations. First, it represents a post hoc analysis of two prospective, observational registries, and is therefore subject to selection bias, residual confounding, and misclassification. Second, treatment exposure was only assessed at baseline; changes in anticoagulation or rhythm control strategy during follow-up were not captured. Third, patients without complete follow-up or with missing data on key variables were excluded, which may have introduced selection bias. Fourth, although our definition of CC was based on pragmatic and clinically relevant criteria, other important domains—such as frailty scores, functional status, and patient preferences—were not assessed. Another limitation of this study is the unequal follow-up duration between the two registries (EORP-AF, 2 years; APHRS-AF, 1 year). We used time-to-event analyses to account for censoring at each patient’s last available follow-up, thereby minimizing bias due to different observation times. However, because late events beyond 1 year were not captured in the APHRS-AF cohort, some residual differences in long-term outcome comparability may persist. Finally, our population is predominantly derived from tertiary centres in high-income countries, which may limit generalizability to broader and more diverse healthcare settings, particularly in low- and middle-income regions of Asia and Europe.^4,49,50^

## Conclusions

CC in AF patients reveals striking geographic disparities. Despite a lower absolute risk of adverse outcomes, Asian patients experienced a stronger relative impact of CC compared with Europeans. Furthermore, these patients were less likely to receive OACs or rhythm-control therapies, suggesting that regional differences in treatment practices may exacerbate clinical vulnerability. These findings highlight the need for increased awareness and the development of context-specific strategies to ensure equitable, evidence-based management of clinically complex AF patients across diverse healthcare settings.

## Supplementary Material

euaf229_Supplementary_Data

## Data Availability

The data underlying this article will be shared on reasonable request to the corresponding author.
