# Supplementary material for: Clinical complexity in patients with atrial fibrillation: exploring differential risk profiles from European and Asian cohorts
Source: Europace. 2025 Sep 19;27(12):euaf229. doi: 10.1093/europace/euaf229 (PMC12722002; doi:10.1093/europace/euaf229)
Supplement: euaf229_Supplementary_Data [file euaf229_supplementary_data.docx]

**Supplementary methods.**

Andrea Galeazzo Rigutini, Tommaso Bucci, Michele Rossi, Enrico Tartaglia,

Amir Askarinejad, Giulio Francesco Romiti, Cecilia Becattini, Giuseppe Boriani,

Hung-Fat Tse, Tze-Fan Chao, Gregory Y. H. Lip

**Supplementary Table 1. Multivariable Cox Regression Analysis for Factors Associated with Primary and Secondary Outcomes.**

| **Variables** | **Composite** | **MACE** | **All-cause of death** | **CV death** | **ACS** | **VTE** | **MB** |
| --- | --- | --- | --- | --- | --- | --- | --- |
|  | **Multivariable**  **HR [95% CI]** | **Multivariable**  **HR [95% CI]** | **Multivariable**  **HR [95% CI]** | **Multivariable**  **HR [95% CI]** | **Multivariable**  **HR [95% CI]** | **Multivariable**  **HR [95% CI]** | **Multivariable**  **HR [95% CI]** |
| **Age** | 1.03 (1.02-1.04) | 1.01 (1.00-1.02) | 1.06 (1.05-1.07) | 1.03 (1.01-1.04) | 1.00 (0.99-1.02) | 1.00 (0.99 – 1.02) | 1.02 (1.00-1.04) |
| **BMI** | 0.97 (0.96-0.98) | 0.97 (0.82-1.14) | 0.97 (0.96-0.99) | 0.95 (0.93-0.98) | 0.99 ( 0.96-1.02) | 0.98 (0.95 – 1.01) | 0.99 (0.96-1.02) |
| **Female** | 0.96 (0.85-1.10) | 0.97 (0.82-1.14) | 0.93 (0.81-1.08) | 0.97 (0.93-0.98) | 0.89 (0.67-1.19) | 1.13 (0.84 – 1.51) | 0.89 (0.67-1.19) |
| **Paroxysmal AF** | 0.80 (0.69-0.92) | 0.91 (0.76-1.09) | 0.66 (0.55-0.80) | 0.58 (0.42-0.81) | 1.25 ( 0.94-1.67) | 1.00 (0.73-1.38) | 0.96 (0.70-1.33) |
| **Heart Failure** | 1.74 (1.54-1.96) | 1.81 (1.54-2.14) | 2.10 (0.81-2.43) | 3.99 (3.05-5.30) | 1.14 ( 0.86-1.51) | 1.38 (1.02-1.87) | 1.83 (1.37-2.45) |
| **Hypertension** | 0.95 (0.84-1.07) | 0.92 (0.78-1.09) | 0.94 (0.81-1.10) | 0.79 (0.62-1.02) | 0.98 (0.74-1.30) | 1.05 (0.77-1.42) | 0.72 (0.54-0.96) |
| **Diabetes** | 1.28 (1.12-1.45) | 1.33 (1.12-1.58) | 1.38 (1.18-1.60) | 1.62 (1.26-2.09) | 1.03 (0.76-1.40) | 1.31 (0.94-1.82) | 1.06 (0.77-1.47) |
| **Thromboembolic events** | 1.38 (1.18-1.61) | 1.55 (1.26-1.90) | 1.31 (1.08-1.58) | 1.40 (1.02-1.92) | 1.18 (0.80-1.75) | 2.15 (1.52-3.04) | 1.48 ( 1.02-2.14) |
| **PAD** | 1.23 (1.03-1.47) | 1.15 (0.90-1.46) | 1.36 (1.12-1.67) | 1.37 ( 0.99-1.90) | 0.76 (0.47-1.23) | 1.13 (0.69-1.84) | 0.96 (0.58-1.58) |
| **CAD** | 1.50 (1.33-1.70) | 1.97 (1.67-2.33) | 1.24 (1.07-1.43) | 1.49 (1.16-1.91) | 5.50 (4.08-7.42) | 0.87 (0.62-1.21) | 0.96 (0.70-1.32) |
| **Cancer** | 2.42 (1.87-3.12) | 1.57 (1.03-2.39) | 2.92 (2.21-3.85) | 1.76 (0.97-3.17) | 0.41 (0.10-1.65) | 2.27 (1.15-4.46) | 3.96 (2.38-6.58) |
| **Dementia** | 1.20 (0.85-1.70) | 1.14 (0.69-1.87) | 1.28 (0.87-1.87) | 1.13 ( 0.60-2.11) | 0.79 (0.25-2.50) | 0.90 (0.28-2.88) | 1.95 (0.90-4.23) |
| **OACs** | 0.57 (0.49-0.66) | 0.50 (0.41-0.61) | 0.59 (0.49-0.70) | 0.40 ( 0.30-0.54) | 0.57 (0.41-0.80) | 0.52 (0.36-0.74) | 0.89 (0.60-1.33) |
| **Asian cohorts** | 0.49 (0.39-0.59) | 0.43 (0.32-0.57) | 0.50 (0.39-0.64) | 0.32 (0.19-0.52) | 0.38 (0.23-0.63) | 0.58 (0.36-0.92) | 0.76 (0.51-1.13) |

**Supplementary Table 2. Univariable and multivariable logistic regression for factors associated with enrolment setting in a Clinical complexity group.**

| **Variables** | **Univariable** | | **Multivariable** |
| --- | --- | --- | --- |
|  | **OR [95% CI]** | **p-value** | **OR [95% CI]** |
| **Age** | 1.03 (1.02-1.04) | <0.001 | 0.98 (0.97-1.00) |
| **Female Sex** | 0.85 (0.72-1.00) | 0.530 | 0.56 (0.44-0.70) |
| **BMI** | 1.00 (1.98-1.03) | 0.772 | 0.84 (0.81-0.86) |
| **Diabetes Mellitus** | 0.96 (0.81-1.15) | 0.674 | 1.76 (1.38-2.25) |
| **Dyslipidaemia** | 0.95 (0.80-1.12) | 0.514 | 1.36 (1.08-1.71) |
| **Smoking** | 1.02 (0.71-1.47) | 0.905 | 1.22 (0.76-1.90) |
| **Hypertension** | 1.14 (0.95-1.37) | 0.158 | 1.46 (1.15-1.87) |
| **Heart Failure** | 0.35 (0.29-0.41) | <0.0001 | 0.54 (0.43-0.68) |
| **CAD** | 0.53 (0.44-0.63) | <0.0001 | 0.67 (0.52-0.85) |
| **PAD** | 0.15 (0.09-0.23) | <0.0001 | 0.17 (0.10-0.30) |
| **CKD** | 0.30 (0.25-0.35) | <0.0001 | 0.56 (0.44-0.71) |
| **Thromboembolic events** | 1.10 (0.89-1.37) | 0.367 | 1.20 (0.90-1.60) |
| **Paroxysmal AF** | 2.51 (2.10-2.99) | <0.0001 | 2.12 (1.68-2.67) |
| **Cancer** | 1.24 (0.83-1.84) | 0.284 | 1.21 (0.72-2.01) |
| **Dementia** | 1.18 (0.79-1.77) | 0.412 | 1.56 (0.84-2.86) |
| **COPD** | 0.36 (0.26-0.50) | <0.0001 | 0.46 (0.30-0.69) |

*Abbreviations: AF , atrial fibrillation; BMI, body mass index; CAD, coronary artery disease; PAD, peripheral artery disease; CKD, chronic kidney disease; COPD, chronic obstructive pulmonary disease; HR, hazard ratio; OR, odds ratio; CI, confidence interval*

**Supplementary Table 3. Univariable and multivariable logistic regression for factors associated with oral anticoagulants use in a Clinical complexity group.**

| **Variables** | **Univariable** | | **Multivariable** |
| --- | --- | --- | --- |
|  | **OR [95% CI]** | **p-value** | **OR [95% CI]** |
| **Age** | 0.99 (0.98-1.00) | 0.141 | 0.99 (0.98-1.01) |
| **Female Sex** | 0.90 (0.74-1.10) | 0.334 | 0.81 (0.63-1.01) |
| **BMI** | 1.00 (0.98-1.02) | 0.772 | 1.00 (0.97-1.01) |
| **Diabetes Mellitus** | 0.75 (0.61-0.92) | 0.006 | 0.80 (0.61-1.04) |
| **Dyslipidaemia** | 0.87 (0.71-1.06) | 0.185 | 1.10 (0.85-1.43) |
| **Smoking** | 0.94 (0.60-1.46) | 0.786 | 0.92 (0.55-1.63) |
| **Hypertension** | 0.87 (0.69-1.10) | 0.244 | 0.93 (0.70-1.22) |
| **Heart Failure** | 0.79 (0.65-0.97) | 0.026 | 0.77 (0.59-0.99) |
| **CAD** | 0.65 (0.53-0.81) | <0.0001 | 0.60 (0.46-0.78) |
| **PAD** | 0.66 (0.49-0.89) | 0.006 | 0.78 (0.53-1.16) |
| **CKD** | 0.98 (0.80-1.20) | 0.873 | 1.10 (0.83-1.45) |
| **Thromboembolic events** | 1.35 (0.99-1.76) | 0.052 | 1.65 (1.16-2.41) |
| **Paroxysmal AF** | 0.68 (0.55-0.84) | <0.0001 | 0.66 (0.51-0.87) |
| **Cancer** | 0.31 (0.21-0.47) | 0.284 | 0.25 (0.16-0.40) |
| **Dementia** | 0.44 (0.29-0.67) | <0.0001 | 0.43 (0.25-0.77) |
| **COPD** | 1.21 (0.86-1.71) | 0.257 | 1.32 (0.88-2.05) |
| **Asian group** | 0.87 (0.70-1.07) | 0.201 | 0.75 (0.57-1.01) |

*Abbreviations: AF , atrial fibrillation; BMI, body mass index; CAD, coronary artery disease; PAD, peripheral artery disease; CKD, chronic kidney disease; COPD, chronic obstructive pulmonary disease; OACs, oral anticoagulants, HR, hazard ratio; OR, odds ratio; CI, confidence interval*

**Supplementary Table 4. Univariable and multivariable logistic regression for factors associated with Rhytmh control strategies in a Clinical complexity group.**

| **Variables** | **Univariable** | | **Multivariable** |
| --- | --- | --- | --- |
|  | **OR [95% CI]** | **p-value** | **OR [95% CI]** |
| **Age** | 0.98 (0.97-0.99) | <0.0001 | 0.97 (0.96-0.99) |
| **Female Sex** | 1.03 (0.866-1.23) | 0.719 | 1.00 (0.80-1.24) |
| **BMI** | 1.00 (0.98-1.02) | 0.772 | 1.00 (0.97-1.02) |
| **Diabetes Mellitus** | 0.70 (0.58-0.85) | <0.0001 | 0.62 (0.49-0.80) |
| **Dyslipidaemia** | 1.07 (0.89-1.28) | 0.454 | 1.23(0.98-1.54) |
| **Smoking** | 0.77 (0.50-1.21) | 0.268 | 0.52 (0.29-0.90) |
| **Hypertension** | 0.96 (0.79-1.18) | 0.749 | 0.95 (0.75-1.21) |
| **Heart Failure** | 0.66 (0.55-0.79) | <0.0001 | 0.77 (0.59-0.99) |
| **CAD** | 0.99 (0.82-1.19) | 0.931 | 0.64 (0.51-0.80) |
| **PAD** | 0.66 (0.48-0.90) | 0.009 | 0.84 (0.58-1.20) |
| **CKD** | 0.94 (0.79-1.12) | 0.482 | 0.92 (0.72-1.17) |
| **Thromboembolic events** | 1.09 (0.87-1.37) | 0.464 | 1.02 (0.78-1.36) |
| **Paroxysmal AF** | 2.35 (1.95-2.83) | <0.0001 | 2.53 (2.02-3.18) |
| **Cancer** | 0.86 (0.53-1.39) | 0.539 | 0.84 (0.46-1.47) |
| **Dementia** | 0.68 (0.41-1.13) | 0.142 | 0.57 (0.25-0.90) |
| **COPD** | 0.67 (0.49-0.92) | 0.013 | 0.75 (0.51-1.08) |
| **Asian group** | 0.78 (0.65-0.95) | 0.015 | 0.53 (0.41-0.69) |

*Abbreviations: AF , atrial fibrillation; BMI, body mass index; CAD, coronary artery disease; PAD, peripheral artery disease; CKD, chronic kidney disease; COPD, chronic obstructive pulmonary disease; OACs, oral anticoagulants, HR, hazard ratio; OR, odds ratio; CI, confidence interval*
